# American Dental Association

**Published:** 1898-01

**Authors:** 


### Reports of Society Meetings.

### AMERICAN DENTAL ASSOCIATION.

(Continued from Vol. XVIII., page 825.)

*August 5, 1897.—Third day.—Morning Session.*

THE meeting was called to order at ten o'clock, by the President, Dr. Truman. The Secretary read the minutes of the previous session, which were approved.

Dr. Taft read the report of the Committee on Necrology, as follows:

This Association can certainly in justice do no less than place upon the pages of its records some token of the high esteem and regard with reference to the work of those who have gone from us this year. The memory of each should be cherished by every member of this Association.

FRANK ABBOTT, of New York, suddenly passed away on April 20, 1897, of cardiac disease, in the sixty-first year of his age. He was a native of Maine, and entered on the practice of dentistry at Johnstown, New York, in 1858, where he practised for several years. He went into the Union army during the Civil War of this country, and was finally taken prisoner; he was subsequently released, and returned to his home, resuming practice for a brief time, after which he removed to New York City, where he established a large practice. He was largely instrumental in establishing the New York College of Dental Surgery, in which he served as Dean until the time of his death. Dr. Abbott was one of the very active members of his profession, interested in all things that pertained to its welfare. As an organizer and teacher he had a national reputation. He was a writer of more than ordinary ability. Numerous papers he presented, read before this body, are ample evidence of this fact. He prepared a work entitled "Dental Pathology and Practice," in which the choicest of these productions is presented. He became an active member of this body in July, 1866, and was very active in promoting its best interests. He was always active in dental society work in his own State. He was a member of the National Association of Dental Faculties, and a member of the Executive Committee of that body. He was enthusiastic in whatever he undertook, and ever ready to give expression to his views; he was kind-hearted, and highly esteemed by all who knew him.

Francis Peabody was born in Boston in 1833. In his early manhood he removed to Louisville, Ky., where he studied his profession with his uncle, Dr. William H. Goddard, one of the most distinguished dentists of his day. He afterwards took a course of instruction in the Ohio College of Dental Surgery, and graduated. He continued his practice in Louisville until 1871, when he went to Rio Janeiro, where he practised for some time, and afterwards returned to Kentucky, where he remained until his death. He allied himself with dental society work early in his career, and was earnest in his efforts to secure the elevation of the profession. He was an active member of the Southern Dental Association, and of many other similar bodies. He became a member of this body in 1888, and was always interested in the work, being present at its meetings whenever his health would permit. For some years he was connected with the Louisville Dental College, as President of its Faculty and Professor of Operative Dentistry. He was a successful teacher, and did much to build up and strengthen that institution. He was a man of marked ability as a practitioner. He was fruitful in devices and methods, a man of generous disposition, whose friendship every one prized.

Dr. S. B. Brown, of Fort Wayne, Ind., was born in 1834, in Vermont, and died June 5, 1897. He grew up in agricultural pursuits. After two years of pupilage as a student of dentistry, he began its practice at Ticonderoga, N. Y. In 1885 he removed to Ohio, and in 1896 to Fort Wayne, where he practised until his death. He received the degree of D.D.S. from the Pennsylvania College of Dentistry in March, 1870. He was much interested in all that pertained to his profession; he was a champion of its dignity, ever ready to come to its defense when it was assailed. During the last two or three years of his life he did much to bring his views to the attention of college classes and faculties throughout the country, by addresses and written communications. He became a member of this body in August, 1869, and was usually present at its meetings. He was of a quiet and rather retiring nature, and perhaps exerted more influence upon his professional brethren by personal contact than by more public efforts. Still, whenever occasion called for it, he was not lacking in the latter. Although he was widely known in professional circles throughout the country, his most marked influence was exerted within the bounds of his own State. He was one of the founders of the Indiana State Dental Society, and occupied its highest positions of trust and honor. He was connected with the Indiana Dental College, either as teacher or member of its Faculty or Board of Trustees nearly all the years of its existence. Who, in Indiana, will fill Dr. Brown's place? He was beloved, and his name will be cherished by all who knew him.

Dr. Eli Slagel was born in 1836, at Fleetwood, Pa. He began the study of dentistry under Dr. W. W. Thompson, and completed his course of study under Dr. Lukens, of Philadelphia. He allied himself with dental societies in his own State, always helping, so far as he might, towards their welfare. He became an active member of this body in 1896. He added many useful improvements to his art, which were highly prized by those to whom they were given. His bearing commanded the respect of all who came in contact with him, professionally or otherwise.

The death of William N. Morrison occurred at Hot Springs, December 20, 1896. He was born in Ohio in 1842. His whole professional life was spent in St. Louis, where he established an eminently successful practice. In his early life he had to contend against great odds, especially when he came to take up his life work. He was a man of great perseverance and industry. In 1864 he graduated from the Ohio College of Dental Surgery. Throughout his career he kept fully abreast with the progress of his profession. He was the first to devise, use, and present to the profession a number of valuable methods, processes, and materials. His interest in the profession never flagged. He was ever ready to receive that which was valuable from his professional brethren, and always as ready to give anything he possessed that might be serviceable to others. He kept up with the literature of the profession, often contributing to it through the journals. He did much to promote association work; he was a member of a number of dental societies, was often present at their meetings, and at such times was ready to do his share of the work. His life presents an example that will influence many in the profession, especially the younger members. He became a member of this body in 1864, and was its warm supporter until the time of his death. His passing away leaves a vacant place that will not be readily filled. The profession and this Association cannot afford to lose such members as Dr. Morrison.

*Resolved*, That the record of this report be placed on a memorial page of the proceedings of this Association; that a copy be sent to the dental journals for publication, and that an engrossed copy be sent to the family of each of the subjects of this report.

(Signed) THE COMMITTEE.

Report and the resolution therein contained were adopted.

*Dr. Taft.*—Your Committee feel sad at the preparation of a report of this kind, not only on account of the character of the men who have passed away, but also because there are so many that have gone within the last twelve months. The report seems voluminous. The Committee is impressed with the idea that what has been said should all be said concerning these men. More would have been said had the Committee followed out their inclinations, but it was not proper to make it too lengthy,—simply to touch upon the most salient points.

The President then called for discussion on the paper and exhibition of Dr. Broomell.

*Dr. Peirce.*—The exhibition I consider was a very successful one, but not one that would call for much discussion. It brought to mind very forcibly the contest of many years ago and recently settled,—the theory of development. I think it may be of interest to give a sketch of the difference between the two theories that were advanced at that time. When Harvey, who originated the theory of the circulation of the blood, had finished that work, and Leeuwenhoek had perfected his microscopic work and placed it before the scientific world, Harvey procured a microscope in 1651, and took it into his laboratory; he also took a hen and twenty-one eggs. Every day an egg was broken and note made of what took place in the ovum. When he completed that investigation, he said, "Syngenesis—already formed—is not true; but epigenesis—to be formed—is true." The clergy and every one told him to keep that to himself and not publish it, and it was buried for one hundred and eight years. In 1759, Frederick Caspar Wolf, of Germany, took the subject up, and said Harvey was right. He went through the same experiments with only one organ—the alimentary canal—and made drawings. Every one then said the animal was already formed in the egg, but Wolf said it had to be developed. Haller came out with a paper and declared it was rank heresy; that every animal was created in the beginning; and he went so far as to state in his paper that in the ovaries of Mother Eve were two hundred billion perfect human beings, and when those were all born, that will be the end of the human family. Haller published that in opposition to the theory of epigenesis. Wolf for many years fought that theory, and it was not until 1789 that he could get a hearing, and get the physiologists of the day to recognize the truth of Harvey's statement. So, to Harvey first, and to Wolf afterwards, are we indebted for the theory of epigenesis,—that the animal is to be developed and is not already developed, and that is the theory that we had so beautifully illustrated last night with reference to the individual organ. It is surprising to know how recently have we recognized the truth in development. You are all familiar with the custom of the Spaniards in giving their children the mother's name. That idea was doubtless the origin of it. The mother only was the parent of the child; the father's traits and disposition had nothing to do with it. The theory was that the father only stimulated development, and that theory held until 1789.

Regarding the exhibition last night, I do not know that those who witnessed it recognize the fact of how difficult it was to show the development of enamel. We had only the space where that reticulum existed. Dr. Broomell tried to get a photograph of it, but the moment he exposed it it contracted. When you saw the development of the dentine, you saw the open space above the cusps, not the enamel at all. The cusp of the enamel was there, but the reticulum that was instrumental in producing the calcoglobulin, that produced the ameloblasts, could not be shown. We have only the space, and it is very rare that we can get the reticulum that is instrumental in producing the ameloblasts. I know the intense and persistent labor of Dr. Broomell in getting these parts in condition to show them. We have never had an exhibition of the kind, giving us the structure in its entirety; we have had only sections and slides. Dr. Broomell is entitled to great praise and commendation.

*Dr. Patterson.*—I was greatly interested in the exhibition, and appreciate the labor that Dr. Broomell has gone through. I do not believe myself competent to say anything further on the subject.

Subject passed.

Section VI., Physiology and Etiology, then presented the following report, through Dr. J. D. Patterson, its chairman:

The etiology and physiology of the oral cavity grows in importance with increased dental knowledge, and of the dentist, who is in reality a professional man, more education in this important field is year by year demanded. In the literature which has come to us during the past year in the annual of the "Universal Medical Sciences," Dr. Sajous, editor, we glean the following upon phagocytosis:

"The controversy as to the part played by phagocytes still continues." Metschnikoff is chief, of course, for the phagocytes, and Pfeiffer chief of the opposition. They each have many supporters. Pfeiffer claims that the destructive processes come "through extracellular action, and that the serum alone exerts bactericidal power;" that the dead germs which he, with Metschnikoff, acknowledges are found within phagocytes were dead when they were surrounded or taken in,—killed by the serum.

Connected closely with this question is the one of "immunity" and what part antitoxins play. Buchner (1896) thinks that immunity is not the result of direct action of the antitoxin upon the toxin, but that both act antagonistically upon the cells of the human system.

Kossell looks upon the bacteria-destroying power of nucleinic acid as playing a part in the development of immunity. Metschnikoff agrees with this, because it gives a part to phagocytes in the accession of immunity. Roux likewise looks upon immunity as due to cell activity. Editor Sajous says, "The preponderance of evidence gives nucleinic acid the greater part of immunity. The question, however, as to whether the acid acts exclusively as a bacteria-destroying agent, or if by its acidity it neutralizes the toxins, remains unsettled."

In the "New Surgery," issued since our last meeting, edited by Dr. Roswell Park, we find on page 312 the following of interest to the dental surgeon: "The study of the bacteria of the mouth is of great interest to the surgeon. The relationship between mouth bacteria and local lesions of the teeth and jaws and mucous membranes—of secondary gastric disturbances—and the study of the mouth as the first point of infection for both local and general diseases is a wide subject. Suffice it to say that normally the mouth contains scores of varieties of bacteria,—saprogenic usually,—but include also common pathogenic forms, which may be found as normal inhabitants of the mouth without injury to the individual; but if the vitality of the tissues is lowered to a sufficient degree, these pathogenic germs may find a favorable breeding-ground for the production of disease. The mouth furnishes a point of infection for most pathogenic germs,—*e.g.*, those of diphtheria, erysipelas, actinomycosis,—the oidium albicans of thrush,—tubercle bacilli, and the virus of syphilis."

In the literature which has appeared since our last meeting, one year ago, bearing upon the subjects of this section, we note an essay upon the "Experimental Study of the Different Modes of Protecting the Oral Cavity against Pathogenic Bacteria," by A. C. Hugenschmidt, M.D., D.D.S., of France (*Cosmos*, October, 1896). An interesting statement in this essay is that after certain oral operations, such as the extraction of a tooth, the soft parts of the injured territory often present the appearance of an ugly wound, and when we think of the pathogenic germs often found in the mouth, it is difficult to understand why such an exposed wound is not more often the seat of severe local trouble, or that it does not bring on a general infection of the economy. There is no doubt that the severe local inflammations which sometimes arise are due to this infection, and the operator should take all precautions. Usually, the phagocytes and the chemotactic and mechanical properties of the oral fluids prevent injury.

We note an article upon "Diseases of the Oral Cavity a Potent Factor in General Diseases," by Dr. S. W. Foster, Atlanta, Georgia, read before the Dental Section of the American Medical Association, 1897, and published in the *Dental Cosmos*, in which the author names the following dental irritations as productive of general diseases:

First, dentition; impacted teeth causing obscure neuroses; destruction of masticating power causing dyspepsia and gastritis; general lack of oral hygiene, which poisons nutrition, causing nervous debility and breaking down of the vital forces; also consumption. Of this last the author says, "It is rare that we see a consumptive in whom we do not find a typical case of pyorrhœa, or at least pus exuding from and around the necks of the teeth, and it seems that the breathing into the lungs of the effluvia from these suppurating sinuses of the mouth might produce this fatal disease."

We note a paper upon the "Etiology of Fetor ex Ore," by Dr. Jerome Allen (M.D., D.D.S.), in which especial attention is called to the fact that the most persistent and lasting fetors are due to the chronic and exudative diseases of the gums, bones, and mucous surfaces. This, we think, will be appreciated by those having pyorrhœa under treatment.

In the INTERNATIONAL DENTAL JOURNAL, December, 1896, is noted an article by William H. Trueman, entitled "Pyorrhœa from a New Stand-Point." In regard to etiology, the author strongly inclines to the belief that the disease is a germ infection.

We note a paper upon "Pyorrhœa Alveolaris," by Dr. George B. Clement, Macon, Miss. Dr. Clement claims that the disease is caused by hypercalcic deposit in the cementum from the cells which form that structure.

In "Pyorrhœa Alveolaris," by Joseph T. Wassell, M.D., D.D.S., of Chicago (*Dental Review*, June, 1897), the writer speaks of its etiology, and suggests a constitutional disorder found in eczema which he believes has an important relation to the affection.

In Roswell Park's "New Surgery" is found the following: "Stomatitis catarrhalis is a simple catarrhal condition resulting from irritation of the mucous membrane from mechanical, chemical, or mycotic causes. It presents the symptoms of desquamating catarrhal condition."

A paper upon "Pyorrhœa Alveolaris," by J. E. Cravens, D.D.S., Indinanapolis (*Dental Digest*, August, 1896).

A criticism of the uric acid theory of pyorrhœa, entitled "Peculiar Pathological Perceptions," by J. D. Patterson, D.D.S. (*Dental Review*, March, 1897).

We further note an essay upon "Stomatitis the Cause of Various Throat Diseases," by Isidore Lett, D.D.S., Philadelphia (*Dental Cosmos*, September, 1896).

In the INTERNATIONAL DENTAL JOURNAL we find an essay upon "Nasal Obstructions which result in Mouth Breathing, with Special Reference to Adenoid Vegetations," by George L. Richards, M.D, Boston. The writer has the following to say upon the etiology of nasal obstructions: "The cause is not very clear; heredity and climate, especially the latter, seems to play a part, as they are found more frequently in the North and on the coast than inland, and to the South scrofula, measles, scarlet fever, and whooping-cough are causative."

An essay upon "Salivary Calculi," by Gustav Fütterer, M.D. (INTERNATIONAL DENTAL JOURNAL), in regard to causation; the author gives proof that in a majority of cases the disease arises from calcic matter being deposited in the ducts. He says, "Small pieces of tartar often break down, and can very easily enter the ducts, especially Wharton's duct, and here we find the most calculi, which are very rare occurrences in Steno's duct. Gravitation will bring the disease down to the bottom of the mouth rather than up to Steno's duct. They will remain there longer, and occasion to enter will offer itself more readily. The same rule holds good in animals." The theory that the uric acid diathesis is causative of the-e deposits is discouraged by the author.

In the *Dental Cosmos* of Feoruary, 1897, appears an article by Dr. M. H. Cryer (M.D., D.D.S.) upon "The Contributions of Dentists to Surgery."

"The Relation of the Teeth to the Ear, Nose, and Antrum," by O. F. Gambati, M.D., Houston, Texas. The particular phase of thought found in this paper may be said to be in the claim that an empyemic condition of the antrum sets up a pathological condition of the teeth.

In the *Practitioner and Advertiser*, July, 1897, the editor, W. C. Barrett, in speaking of the etiology of "Diseases of the Maxillary Sinus," says renewed attention has been directed to the fact that a diseased condition of the frontal and other sinuses is sometimes present, thus preventing diseases supposed to be wholly in the antrum. When the case is obstinate, it is recommended that other cavities which may drain into the maxillary sinus should be exposed."

In the *Dental Cosmos* of February, 1897, James S. Williams, M.D., D.D.S., relates a case of "Herpes Zoster, associated with Dental Trauma," in which the eczema appeared upon the left temple and involved the eye, caused by a diseased left superior cuspid, and which trouble disappeared upon the extraction of the tooth.

"Cancer of the Lip," by C. L. Gibson, M.D., New York City (*Dental Cosmos*, June, 1897). The causation of this disease is analyzed as follows: "As regards the cause of this disease, to traumatism in general is ascribed the chief place. The lips are easily abraded, cracked, chapped, burnt, and otherwise maltreated, so that abundant sources of irritation are furnished." As regards the element of heredity, there is an ever-increasing tendency to belittle its importance. It is undoubtedly true that the belief in heredity, so strong until recent years, was based to a great extent on tradition. The strong efforts which we are all making to place medicine upon the plane of an exact science discourages the acceptance of vague theories and histories concerning cancerous relationship, and, subjected to an exact scrutiny, the family history of cancer is reduced to so small an extent as to be looked upon only as an interesting coincidence. The author also points out that it is startling to see how many cases of lip cancer have been "acquired by living in dwellings previously occupied by the subjects of cancer."

"Dentistry and its Relation to Medicine" is the subject of a paper by Archibald Dunn, M.D., in the *Dental Practitioner and Advertiser*, April, 1897, in which the author cites two recent cases in his practice, one in which hemicrania was caused by an obscure cavity in the buccal surface of a superior third molar, and one in which convulsions and epilepsy had causation in a filled lower molar.

Dr. Patterson further reported that the officers of the Section have tried to secure papers in addition to the report of the chairman, but have been unsuccessful.

Section VII., Anatomy, Pathology, and Surgery.

*Dr. Barrett.*—I would say that I have not the written report with me at present, but will send it in later to be published in the transactions. The first part of the report refers to the subject of anatomy. It has been urged that there is nothing new to present, because there have been no material changes in the anatomy of the human body within the past generation or two; but there has been a vast change in our comprehension of the human anatomy. Dr. Cryer, two years ago, exhibited specimens, and he showed that the profession in the past and the writers and authors on anatomy have been in error; that they have not the truth of the matter, and that the infundibulum was really in many cases opening into the antrum itself, and hence degeneration might take place in the frontal sinus, and the pus discharged from that might ooze down and become a source of irritation in the antrum. Hence, in trouble of the antrum it would be almost impossible to diagnose it clearly.

There is another point in anatomy which I think is of importance to us, and that is this: Books on the subject represent the pulp of the tooth, the nervous supply of the pulp, as having been received from the internal dental artery. I believe that to be a positive error. The proof that this is not the case lies here: that no continuation of the artery or nerve can be traced beyond the pericementum, and it seems to me that the pericementum is the central organ which supplies the pulp; that the foramen of a tooth is a delta, spreading out, dipping down, and uniting with the pericementum from which it receives its supply.

It has been a question how and when it was possible to cap a pulp successfully. I believe that to be solved by this one fact in anatomy. There are no lymphatics within the pulp to take up any matter that shall have passed off. As in the brain, because of the change of the character of the blood-vessels and the lack of absorption, so in the pulp, which is surrounded by the tooth itself, as the brain is surrounded by the walls of the cranium; there are no more absorbents in the dental pulp than in the brain. That gives us a diagnostic sign beyond which it is imprudent to proceed. There is no connection between the pulp and the internal artery for the blood-supply.

I would refer to some new operations by Dr. Brophy for congenital cleft palate, the operation to be performed at the earliest possible moment after birth. The strangest thing is that when you bring these two parts together there goes on development. It seems to develop as perfectly as any other.

We cannot call ourselves scientists as long as we pay no attention to any part of the body except that which we cultivate for our own special benefit. It is our business to study the various relations of the teeth to the other portions of the body. What shall we have to answer if another part of the scientific world steps in and occupies that place for itself? I have secured the co-operation of Ward's Natural Science Museum, at Rochester, and early in the year they commenced to gather together material for exhibition here. The exhibition is offered for sale, and is intended to be sold as a whole; it should not be separated. It should go to some college.

The Section desires to present the following papers:

"Report of a Case in Practice: Operation for the Opening of the Frontal Sinus and Infundibulum," by Professor Truman W. Brophy.

"Further Studies of the Relation of the Frontal Sinus to the Antrum," by Professor Thomas W. Fillebrown.

"Structural Development," by Professor C. N. Peirce.

Also a volunteer paper by Charles H. Ward, entitled "The Teeth as a Factor in Anthropology."

The Committee desires to present the catalogue of the collection, which it has had printed. It makes a pamphlet of thirty-two pages, although we tried to condense it as much as possible. We suggest that it would be well to publish this catalogue in connection with the report, that the world may comprehend something of what the American Dental Association is doing.

Dr. Taft moved that all the papers be heard without discussion, unless there should be time to discuss the same.

Motion carried.

Dr. Brophy and Dr. Peirce then read their papers, after which Mr. Ward's paper and that of Dr. Fillebrown were read by title only.

Section passed.

Section I., Prosthetic Dentistry, Chemistry, and Metallurgy.

Dr. Waters reports that there is one paper, the title of which is "Opening the Bite with Cap-Fillings, Preserving the Vitality of the Pulp," by Dr. M. F. Finlay.

Dr. Finlay then read his paper, as follows:

The patient, male, aged sixty-two years; every tooth present in upper jaw, and all in regular position, with the exception of left cuspid, which closed inside its opponent of the lower jaw; the four incisors were very much worn, so that the palatine surface presented a straight line or inclined plane from labio-incisive edge to palato-gingival line. These same incisors when jaws were closed came in contact with and pressed upon the lower gums. The bicuspids and molars of the left side showed effect of mechanical abrasion, and the outer cusps lapping pretty well over the outer or buccal side of the lower teeth opposite, the bicuspids and molars of the right side showing scarcely at all. On the lower jaw, the first and second molars, right side, were lost, accounting for the last-mentioned condition of the upper jaw, at the same time throwing the work of mastication almost entirely upon the left side of the mouth, and in consequence causing the wear spoken of on the teeth of that side on the upper jaw, the lower teeth suffering more loss of tissue than the upper ones, and they also being, as it were, driven down into their sockets from the undue pressure, as appears in the model.

The lower incisors were worn to an inclined plane, presenting a surface opposite that of the upper incisors,—the plane running from the palato-incisive edge to the labio-gingival line, and the incisive edges impinging on the upper gums when teeth were closed. My belief is that quite a good deal of responsibility for the excessive wear of the teeth, and the depressed condition of the lower teeth, left side, is due to the malposition of the left upper cuspid, one of the main abutments of the arch being so misplaced as to offer no resistance to the settling together of the two jaws, whereas if the cuspid had been in its normal position, or had been forced there by regulating at the proper age, it would have been quite impossible for such a condition to have attained. The contact of the front teeth with the gums opposite in closing the mouth, and the close proximity of the outer cusps of the upper molars of the left side with the gums of the lower jaw, forcing the food in the process of mastication in painful contact with said gums, caused the patient so much discomfort that he sought relief, or at least advice, as to what might be done to the best advantage to obtain the same.

After taking impressions and getting models, my first thought or inspiration was to open the bite with cast or struck-up slabs of metal or cap-fillings, as I have decided to designate them. How to attach the cap-filling was the question to be solved. In consulting several of my brother practitioners, all at first seemed to oppose my plan as not being at all feasible. First thoughts or impressions are the most lasting, and there was no exception to the rule in this instance, as I persisted in my belief that a way could be found to attach these cap-fillings which would be satisfactory alike to patient and operator.

My reason for not crowning the teeth, as was advised by some, was that too much tooth-structure would have been sacrificed upon the approximal surfaces in preparing for such a procedure to satisfy my conscience. The teeth were not decayed or darkened, but were worn down; also all the teeth had living pulps, and I was afraid to disturb the relation of the gums to the necks of the teeth as much as would have been required to place crowns over three adjoining teeth, with no tooth missing to afford easy access to any of the surfaces to be cut away.

Now for my plan of procedure: The second bicuspid and the first and second molars on the left side, lower jaw, were restored or built up with these cap-fillings, and the second bicuspid and third molar on the right side, lower jaw, were crowned to carry a bridge to replace the lost first and second molars, the occlusion raised to correspond with the open bite.

In preparation for these cap-fillings, the occlusal surfaces of the teeth to be capped were ground, so as to make them nearly a plane, although not absolutely so; then, taking an impression in moldine and making die and counter with Melotte's metal, I struck up pieces of nearly pure gold about 32-gauge, to fit these ground surfaces, and drilled four holes in the molars and three in the bicuspids, punching holes in the little pieces of gold to correspond in position and soldering platinum pins in them, and then united to them cusps struck up on a die plate, and filled those, making a cap of considerable thickness.

I ought also to say that the first piece of gold stamped to fit the tooth was thickened up, a second piece stamped over the first and united to the first before the pins were soldered in. Twenty-carat solder was used in fastening the pins and filling the cusps, and the two pieces united with eighteen-carat solder.

As to the location of the pin-holes in the molars, I drilled them at a safe distance from the margins of the occlusal surfaces of the tooth in the centre of the four sides, by this means avoiding the cusps, and thereby also the cornea of the pulp, for, as above stated, all the teeth had living pulps.

As a result of my labors, the second bicuspid and first and second molars of the left side, lower jaw, were raised with cap-fillings, the approximal surfaces of the teeth not being disturbed in their relations one with the other, and the second bicuspid and third molar of the right side made to carry an all-gold bridge, thus raising the bite and restoring the lost masticating surfaces, preventing contact of the front teeth with the gums, and opening the bite sufficiently to prevent pain during mastication. The cap-fillings were fastened with cement, as was also the bridge, and all set at the same time, so as to avoid irritation by pressure on a single tooth or one side of the jaw.

As an incident in connection with the construction of the bridge, when working on the third molar, the patient related his experience in having had a gold filling put in on the buccal surface of this tooth by one of our late esteemed friends in New York City, which required more than a year in the preparation of the cavity and insertion of the filling, and for which he paid two hundred and fifty dollars. Since that occasion he said he had often wondered for what purpose he had preserved this third molar, as it was really of not much use to him, the two teeth in front of it having been lost, but now he realized why it had been preserved to advantage, because it enabled him to have this bridge put in, using the tooth as one of the abutments.

Dr. Finlay presented with the paper models and photographs of the case.

DISCUSSION.

*Dr. Peirce.*—I want to make allusion to one item touched upon, and that is the matter of tartar or salivary calculus. I think the dental profession has been quite lax in expressing that properly. Nearly every teacher has spoken of it as an amorphous deposit. It has been my fortune to make a number of experiments in the past year trying to ascertain whether the tartar upon the teeth was simply a precipitate of the lime in the saliva, or whether its deposition was due to organisms in the saliva. A quantity of saliva placed in a vessel outside of the mouth, although it may be saturated with lime, will not deposit that lime in the vessel, but if we can place in the saliva organisms such as the leptothrix, I am satisfied we can get a deposit on the sides or bottom of the vessel.

I believe the deposition of lime upon the teeth is due not only to the fact, which is essential, that there must be lime in the saliva, but also to the fact that we have organisms which are instrumental in its deposition. I took two patients, in whose mouths there was a rapid and profuse deposition of tartar upon the teeth. I asked them to keep their mouths as pure as possible with a strong antiseptic for a limited time. The care exercised in washing that mouth or keeping it saturated with a strong antiseptic without interfering at all with the natural secretion of the saliva from the glands prevented the deposition of calculus on the teeth. That satisfied me that the deposition was not only due to the presence of lime in the mouth, but also to the organisms in the mouth.

Professor McQuillan prepared a paper on the subject of tartar in the mouth being a result of some organism, but he did not sustain his theory, because he could not discover that organism. Microscopic investigation did not discover anything akin to the coral organism. If any gentleman here will take a patient from whose mouth he is in the habit of removing tartar, and have the patient use a strong antiseptic, the tartar deposited will be found to be of a minimum quantity. I use boracic acid and gaultheria. It destroys the micro-organisms, and prevents them from depositing tartar on the teeth. Salivary calculus is due to an organism as well as the lime in the saliva.

*Dr. Barrett.*—I cannot agree with Dr. Peirce, for two or three reasons. How can a micro-organism produce any kind of deposit or any kind of change? Precisely as a potato decomposes the ground in which it is, so the micro-organism decomposes the medium in which it grows and excludes certain of the atoms of oxygen or carbon, taking out whatever it wants for its own organism; these excluded organisms make new organisms and produce the characteristic by-product. That may be a ptomaine of such exceeding power and strength that we have scarcely any conception. Take a single ptomaine and compare it with any of our poisons. One-millionth of a grain is poisonous in the bacillus of tetanus. This is always, so far as I know, in the presence of organic matter. The coral is referred to, but it is built up in inorganic matter; that changes the relation. The change that takes place is utterly different in calcic material from that which takes place in inorganic matter. In the presence of albumen, calcic matter assumes a definite, concentrated form, and it becomes the calcoglobulin of the salts of the tooth. In inorganic matter it is different. We have the carbon dioxide of the breath meeting the saliva, which contains the lime, and in its presence there is an amorphous precipitate, and so the tartar is amorphous in its character.

*Dr. Taft.*—This is a new thought to some, in regard to the action of organisms in the precipitation of calcareous material upon the tooth. When the saliva passes out of the salivary ducts its environment is changed. Its temperature is lower, perhaps, especially if the mouth is kept open. It comes in contact and is mixed with the mucus of the mouth, which changes its chemical character, and it is in contact with the atmosphere, and probably absorbs oxygen from it. This is supposed to be sufficient to cause a precipitation or a dropping down of the calcareous material in the saliva, which has been held in solution. When it comes into the mouth and passes out into the ducts and oral cavity, the conditions and environments are so changed that it is no longer able to hold this material in solution, and it is precipitated. Saliva contains carbonic acid gas, and that is neutralized after the fluid passes into the mouth. This is an important factor in the retention of lime salts in solution. In vessels in which lime-water is boiled you find that rapid precipitation takes place. The precipitation in the mouth is not very rapid; it requires weeks, months, and sometimes years, to produce any considerable accumulation. Usually it takes months, although there are exceptional cases, of course. The more dense varieties require a long time for the precipitation. Dr. Peirce referred to the use of antiseptic agents in the mouth to minimize or destroy this action. The material is used principally with a brush, is it not?

*Dr. Peirce.*—No; usually as a wash in the mouth.

*Dr. Taft.*—May not that agent which you have used so change the saliva as to reduce its solvent power upon this material, and it therefore lets go of the material which is held in solution, and that is thrown down? A large share which is precipitated in the mouth does not lodge on the teeth, but only in those places where there is no friction, or action, to keep it away and get rid of it. It had not occurred to me that organisms play any part in this matter, but we would do well to investigate it.

*Dr. Patterson.*—The matter of the calcic deposits was not brought up in the report at all in this way. I would hesitate for a long time in believing in the theory put forth by Dr. Peirce. I think he starts upon a proposition which is totally wrong, that in the solution containing these inorganic materials we will not find a precipitate. There is not a winter passes that I do not make the experiment right before the class to which I lecture in our college.

I have made that experiment so many times and found the deposit on the rods which I place in the solution that I think he is on the wrong basis.

As to the micro-organisms, I do not see how that is possible, except on account of their presence, not on account of any of their products.

The subject of etiology is a very interesting one to me, and I have been discouraged, in trying to make this report, that I have not secured the assistance from those to whom I appealed. It was a very interesting question to me, especially on account of some local experience we have had. There have been six suits for malpractice against dentists in the State of Kansas. Only last week one of my friends was mulcted in the sum of six thousand dollars. The question that the jury had to determine was, what caused the trouble in the jaw, resulting in the loss of teeth and the loss of process? The damage in one case was given on account of the belief that the hypodermic needle which was used for anæsthetizing the parts was infected. The question went over the various theories, from physicians to dentists, in regard to the etiology of the trouble. It has been very interesting to me, but I do not know what we will do if ignorant juries are to decide upon what causes certain trouble and certain results.

Subject passed.

*Dr. Patterson.*—The Auditing Committee report that they find the accounts of the Dental Protective Association correct.

*Dr. Fillebrown.*—As the result of conference with many members of the Southern Dental Association, they are desirous that we meet with them in the pavilion of the Hygeia Hotel, so the Committee has consented to a change in the programme in regard to meeting there instead of here.

Adjournment.

*August 5, 1897.—Third Day.—Evening Session.*

The meeting was called to order at half-past eight by the President, Dr. Truman.

The minutes of the previous session were read and approved.

*Dr. Peirce.*—Under the head of miscellaneous business, the Executive Committee has a few things to present. The supposition at the present time is that the American Dental Association will terminate its present existence with this session. If so, we need to make provision for the publication of our transactions. Shall the transactions be referred to the Publication Committee, with authority to publish them, as previously?

Dr. Smith moved that the transactions be referred to the Publication Committee and published, as usual.

Carried.

The second item is that the Secretary has on hand a number of copies of the late transactions. Shall they be given to the members, or shall an effort be made to preserve more than the specified number? Some years ago a motion was made that the Secretary retain in his possession ten copies of each publication. I understand the Secretary has many more than that on hand. Shall those ten from each publication be kept for the benefit of the members of this Association? It seems to me desirable that we shall keep on hand a limited number of our publications, because in the future there may be some demand for them as a matter of history.

*Dr. Donnelly.*—The National Museum at Washington will take care of anything of that kind in any way you desire. If you want them preserved without being used, there is storage room for them; if you want them put on the shelves, they will be placed there for reference by dental students; if you want them given to libraries or exchanged with other societies, correspondence to that effect will be opened at once.

*Dr. Peirce.*—I move that those copies be given in charge of the Army Museum and Library in Washington, and that an order be drawn on the Treasurer for the expense of placing them in that position.

*Dr. Fillebrown.*—I had the pleasure of meeting Dr. Huntington, who is connected with that Museum, on my trip here. I think no better disposition can be made of our proceedings than to send the copies there and let them use them for the best interests of the world at large.

*Dr. Peirce.*—I would move also that the very valuable book now in our possession be disposed of in the same way,—the old book which contains the names of the founders of this Association. It should be rebound and placed where it can be protected for all time. I should add to that list the names of all the present members, together with the date of their coming into the society, as a matter of history and record. It would be some little expense, but our treasury can afford it.

Both motions carried.

Another matter is as to the funds of this Association. Provision was made this afternoon, before the new association, that we should pay into the new association a certain sum of money,—equal to one dollar for each active member of this society. We will still have on hand fifteen hundred dollars or more, and it is for this body to state what disposition shall be made of this fund. The question is also raised, Shall we pass a resolution to disband this Association after the present session is through?

*The President.*—We are disposing of money and everything,—making our will, as it were. I think the first real business would be to decide what shall be done with the Association.

*Dr. Crouse.*—I do not think many members understand this matter, and I move that at the adjournment of this Association to-morrow we adjourn *sine die*, except such work as is necessary to be completed by the officers to close up the Association's affairs.

*Dr. Crawford.*—From what has been said, it occurred to me that it would be the proper thing to publish the transactions of the two societies together,—the American Dental Association coming first and the Southern Dental Association last,—so as to put in one volume an authenticated historic account of what has been done.

President Truman left the chair to speak from the floor.

*Dr. Truman.*—As I understand this proposition, we are to form three organizations, at least, in the United States. The Southern Dental Association propose to hold their organization as one of the branches. The Pacific Coast Congress expect to make that a branch. Why is it necessary that this American Dental Association, fully organized, with all the necessary accompaniments of an association, should disband? It strikes me that we should retain the name of the American Dental Association, Eastern Branch, and continue our work. We must reorganize a branch in the East if we do not. Why should we throw all this away? It seems to me a very loose way to do, and I cannot comprehend it. I left the chair reluctantly, because no one seemed to have that idea. I hope that will be considered before we plunge into obscurity. If the Southern Dental Association propose to keep their association going, why should we disband and be swallowed up entirely in that national organization? I do not feel that that is the correct thing to do.

*Dr. Taft.*—Speaking of the publication of the proceedings, it strikes me that the proceedings of this body might be published, following them with the proceedings of the Southern Dental Association, and then follow those two with the proceedings of the joint body; then we would have them altogether. I suppose the proposition in mind, when this organization was effected, was that the American Dental Association and the Southern Dental Association would pass out of existence; there would be no longer any occasion for them. That was the feeling I had, and the sooner the idea of continuing them is abandoned the better. I see no occasion for these bodies remaining any longer. The Southern Dental Association had some little work to do, which they intend to accomplish within a year or two, to which, of course, there is no objection; but I do not know what work the American Dental Association has to do. If you continue this, you simply make another organization instead of uniting and consolidating the profession into one association that shall be far more out-reaching than either of these could be separately.

*Dr. Truman.*—Was it not suggested that there should be an Eastern branch formed?

*Dr. Taft.*—It was suggested to have branches, but here is a point, What shall be the mode of operation of these branches? This consolidated body will meet one year at one district, the next year in another, and the next year in the other. It is intended to be an annual meeting.

Dr. Truman resumed the chair.

*Dr. Taft.*—I heard these resolutions and rules read twice, and endeavored to follow them, and the idea I obtained was just as I have stated,—that there would be a consolidated body and that this Association would pass out of existence, and that the same would be true in regard to the Southern Dental Association. I consider that it would bring the forces of the profession all together and make the work more efficient, bringing the people of the different sections of the country together in unity, harmony, and accord for the promotion of the profession. If you will have three or four different sections all over the country, what will be accomplished?

*Dr. Fillebrown.*—Our movement in making a new association does not, in any practical sense, obliterate this Association. It is simply a change of name. These same men, with an enlarged membership, meet next year exactly on the same relations to the country that this Association does to-day. Why should the American Dental Association lose its name and the Southern Dental Association not its name? The Southern Dental Association is a local organization, and its membership does not extend beyond the limit of the Southern States. The American Dental Association is a national association. There are to-day forty-seven members in the South belonging to the American Dental Association; that is more than there are in the West. We are simply changing our name and going ahead. The Southern Dental Association is already organized as a profitable branch. I think branches will exist. They have existed in the British Medical Association and the British Dental Association for many years, and profitably, and probably will continue so. The Southern branch will meet perhaps next year in Florida and hold a meeting; then the members will come and meet with the National Association in the summer. I hope I can relieve our President of his apprehension that we are dying out. I hope this resolution will prevail. I like the idea of Dr. Taft, that we publish our proceedings in the same book, and that they shall be interchanged. I think it would be a very nice thing, and in it all the proceedings of the new body could be published.

*Dr. Taft.*—The idea of endeavoring to copy after the British procedure is hardly possible. Why should not all the State societies be branches and send delegates to the Association?

*Dr. Donnelly.*—If I understand Dr. Truman's point, it is a very good one. This country has been divided into three sections. In one section the machinery is already in operation for the organization that is to represent that section. In the West there is the Pacific Coast Association, where the machinery is also in operation, and will continue under the same name. This Association has, to a large extent, been an Eastern association, not in the sense that it is not American, but in the sense that the men composing its membership are mainly from the East. That section is left without an organization, and without the machinery to put it in motion, and Dr. Truman's suggestion seems to be a good one, in that he calls our attention to the fact that there must be an organization for that section started anew. Why not use the machinery that we have and keep it in motion to form an organization to represent that section?

*Dr. Patterson.*—It seems to me the whole intent of the consolidation would be frustrated if we continue the American Dental Association. If we keep up ours and the Southern Dental Association theirs, we would be only forming another organization.

Resolution carried.

*Dr. H. A. Smith.*—I just want to call attention to the fact that this is a national body, and it would be in bad taste to maintain this organization with its present name.

*Dr. Peirce.*—The officers now have authority to transact all the business essential to the closing of this organization and to pay all bills pertaining thereto. The question now comes, What shall be the disposition of the funds remaining on hand after all has been accomplished that is authorized in the resolution adopted this evening?

*Dr. Crouse.*—I have had my eye on the funds that would be left over in this Association for some time. The members of the Dental Protective Association know that without assessing the members I have a debt on my hands to pay. To my mind, the proper thing for this Association to do is to donate this money and transfer it to the Dental Protective Association to help pay that debt. I can save the Protective Association one thousand dollars if I get enough money together to pay our attorneys. I have hoped the membership would be increased or some way would be found to pay the debt. I can, of course, sell off our securities and pay the debt, but everybody says we must not let the Association go down. I have asked for donations from the members, and have received one thousand eight hundred and twenty-nine dollars altogether to pay an indebtedness of several thousand dollars.

*Dr. Fillebrown.*—I move that any residue of funds of this Association, left after discharging all of its obligations, shall be turned over to the Dental Protective Association.

Motion carried.

*Dr. Fillebrown.*—I move that the Treasurer of this Association pay into the treasury of the National Association an amount equivalent to one dollar for each permanent member of this Association.

Motion carried.

Dr. Donnelly read the report of the Committee on Library, and the resolution accompanying the same was adopted.

*Dr. Donnelly.*—I do not want to have to go to a man and urge anything of this kind on him. If it does not strike him so as to enlist his sympathy and his enthusiasm and his strong moral support, I do not want to see the work attempted at all. I have for years had this matter before me constantly, and that resolution embodies an idea that I had when the matter was first spoken of, that the dental profession is not represented in any part of the government. There we can put a man whose life may be devoted exclusively to the subject,—some good all-around man, at such a salary as they pay medical men who do similar work for the medical profession. It requires endorsement, however. Dr. Fillebrown urges us to go ahead, without asking you to speak on the subject.

Since he met one of the gentlemen connected with that department he has exhibited more interest in the matter than he ever had before; and if you were all to put yourselves in touch with the matter, much more would be accomplished through that institution.

*Dr. Truman.*—Does the government propose to pay the salary of such a man as you mention?

*Dr. Donnelly.*—The Committee is of the opinion that it can secure the employment of a suitable person at the expense of the government, at such a salary as they pay to men who do like work for the medical profession. This Association has endorsed the Museum as its national museum. Whatever is done now is done in the name of the American Dental Association, unless the National Dental Association takes it up also. We have gone so far, and this Association should have the credit of continuing the scheme to a definite end.

Section II., Dental Education, Literature, and Nomenclature.

*Dr. Ottofy.*—The Section desires to file its report without reading same. The portion on nomenclature has been prepared by Dr. Guilford.

Report received and referred to the Publication Committee.

Section III., Operative Dentistry.

*Dr. Crouse.*—The only work has been on the subject of amalgam and its treatment. Before coming here I had the Secretary write to the officers of the sections that if they would procure me specimens of the different amalgams in use we would make practical tests here. We got about eighteen or twenty samples of amalgam. The work is necessarily incomplete, as we worked only two days on it, and some of the amalgams require a longer time to show what their movements would be. I thought I would take the responsibility of showing something of what we are doing without giving the results in detail. It is a question whether this work is developed far enough for the section to recommend the publication of the makes of amalgam tested. They are not very favorable, although the experiments have hardly gone on far enough to know exactly what they will do. I have prepared a short paper in connection with these investigations, illustrating somewhat the line of work that I have been doing. The steel tubes which I will pass around are all of the same size and shape. The amalgam is packed into them, and a little steel button placed in there before the amalgam is hardened, and then a reading taken on the micrometer. I have watched it by the hour, because the action has been so strange and so fascinating to me. I have here Dr. Black's dynonometer, also Dr. Wedelstaedt's. This instrument we used for testing the strength of the blocks, that is, how much stress they bear before they break to pieces; also to put blocks under this metal instrument, and, placing sixty pounds pressure on them, let them stand and see what the flow was. The question of flow is not much understood. It means the changing of the form of the mass, not breaking it, but bending it or changing it gradually under a sixty pounds pressure. Many of the amalgams showed that they are absolutely worthless where any force comes on them. I have several dies, and I have different operators make blocks with the same amalgams, and I have never found an operator that worked uniformly. They mix their own amalgams and mix them as they please. The working of amalgam is also an important item which we do not understand. It is a great wrong to the dental profession not to have the best amalgam and some form of testing it.

Dr. Crouse then read his paper, entitled "The Amalgam Question," as follows:

The exact date when a combination of different metals reduced to a plastic state with mercury was first used as a filling-material in decayed teeth is not known to the writer; but the fact that amalgam has been a subject of constant dispute for the last thirty-five years, and that frequently during this time these controversies have been so vigorous as to destroy personal friendship and rupture the harmony of dental associations, and the additional fact that the questions under dispute are not settled, is sufficient proof that there are elements of uncertainty about amalgam and its use in dentistry which are more than trivial.

My use of amalgam has been very limited, on account of the unfavorable results from its use by all classes of operators. Whether performed thoroughly or in the most careless manner, the fillings of amalgam in the mouths of the human family are far from satisfactory. The fact that the ravages of decay in many mouths continue and make fresh attacks beyond good fillings, where the tooth substance is left exposed, and the causes which induce decay in the first instance are still present, prevents good fillings and good filling-material from being distinguished from poor and imperfect fillings and worthless materials. Even good gold fillings in unfavorable conditions often fail to accomplish what was expected, and this fact prevents a complete detection of careless and imperfect operations when this one reliable material is used.

From this line of reasoning, what is the stimulus to encourage the best efforts when using what ranks as a second-best filling-material employed by the dental profession, when out of sixty-odd different preparations of alloy accurately tested not one met all the requirements for a good filling-material? These faults and defects have always been known to exist, and efforts have been made to detect which one of the great number of amalgams offered for our use was the least objectionable, or, stating it in another way, which are the best offered. Filling cavities in ivory tooth-brush-handles, glass tubes, etc., and then immersing in some colored fluid which would indicate the leakage, and various other tests have been employed; but we are indebted to Dr. Black for the first scientific method of testing amalgam; by the invention of a micrometer and dynonometer he was able to take accurate measurements and discover something of what occurred after amalgam was mixed and placed in cavities, and determine scientifically their value; also, to some extent, what changes took place in amalgam, fresh-cut, and after it had been what is known as "aged." The dental profession is indebted to this scientific investigator for many valuable facts brought out and many fallacies corrected, but we feel justified in the statement that the profession owes him a debt of gratitude for this work more than any other.

While I have been able to confirm many facts brought out by Dr. Black, it is not my intention to review what he has done, as it has already been published; but rather to supplement, and with familiar instruments to take up other lines of work which are much needed:

*First.* To make tests of amalgams now in general use, and give the results to such as are entitled to them. This I trust will prove beneficial not only to us but to our patients as well.

*Second.* To show by the same aids that we do not know how to use amalgam when a good one is furnished; and I hope thereby to get more thought and skill developed in its use. I have gone far enough in this work to make a radical statement,—namely, that the manufacturers are putting together metals without the necessary equipment or the essential knowledge of the laws of metals and their relation to each other. At any rate, I will agree to prove that the amalgams on the market, as a rule, are unreliable and not uniform, lacking the essential qualities for a good filling-material. The study of the question has so absorbed my thoughts and roused my energies that I can no longer hold them in check. Let me present the picture to your view. A profession that boasts of its accurate attainments, and which has the reputation of having made more rapid advancement than any other, is without any means of knowing the quality or behavior of the material when placed in the cavities of the teeth that is more used by it than all others combined. Hence the dentists at the present time are almost helpless in making a selection from the variety of amalgams in the market, the choice being entirely guesswork with them. I make this statement with evidence to sustain it beyond question of doubt, for the tests of amalgam cannot be made either in or out of the mouth without an elaborate and expensive outfit and an expenditure of much time and careful work, so that in making the selection of amalgam a simpler method must be found. Since starting these investigations I have had unusual opportunities to ascertain what amalgams the profession were using, and I have found some of the very poorest in the hands of the best operators. In one brand especially it would be hard to find any less desirable qualities; yet this amalgam I found in more first-class dentists' offices than any other. The answer from dentists why they had selected this or that amalgam demonstrated the fact that they had no idea what working qualities it must have to be fit for use. Most practitioners demand that an amalgam should be very plastic to be easily mixed, and require but little mercury; yet the exact opposite of these qualities characterize a good alloy. The use of plastic amalgams is a great mistake, for it is impossible to pack it perfectly in a cavity. This is readily demonstrated by manipulating under a magnifying-glass. The mass will be seen to move or shift from one side to the other, working like a molten mass.

Another mistaken notion is that an alloy is injured by manipulation after it commences to set. The exact opposite is true, for a stronger and better mass can be made if the amalgam be properly handled and put to place by heavy hand-pressure, or by malleting after it has fairly begun to set. This may not hold true with all varieties, since my experiments and investigations have been confined to one preparation; but I believe it will be found true as a general rule.

The third fallacy accepted by so many is that the least amount of mercury possible to make the mass pliable is the correct manner of mixing; but in this case again the opposite is true. The greater amount of mercury that can be incorporated into an alloy without squeezing out any surplus when reasonable pressure is applied the stronger the filling will be. An amalgam which is easily mixed is generally recommended, when exactly the opposite is evidence of a good alloy.

I have brought with me the micrometer, two kinds of dynonometers, and other implements, and invite inspection and practical tests with the variety of amalgams I have procured. In this way I hope to arouse more than usual interest in this important subject, although all tests cannot be completed, as some amalgams will shrink and expand so that the reading will not show changes for many days, and the blocks of some will not harden enough to test their exact characteristics as to stress and flow in twenty-four hours, or even in forty-eight hours. However, many essential qualities may be shown while we are here, and I hope the benefits gained by those listening and taking part will be as great as the pleasure I have derived from this work.

The discussion of this subject was made the first order of to-morrow morning's business.

Adjournment.

(To be continued.)

